# Mitochondrial Mutations in Adenoid Cystic Carcinoma of the Salivary Glands

**DOI:** 10.1371/journal.pone.0008493

**Published:** 2009-12-30

**Authors:** Suhail K. Mithani, Chunbo Shao, Marietta Tan, Ian M. Smith, Joseph A. Califano, Adel K. El-Naggar, Patrick K. Ha

**Affiliations:** 1 Department of Surgery, Division of Plastic and Reconstructive Surgery, Johns Hopkins Medical Institutions, Baltimore, Maryland, United States of America; 2 Department of Otolaryngology - Head and Neck Surgery, Johns Hopkins Medical Institutions, Baltimore, Maryland, United States of America; 3 Johns Hopkins Head and Neck Surgery at the Greater Baltimore Medical Center, Milton J. Dance Head and Neck Center, Baltimore, Maryland, United States of America; 4 Department of Pathology, University of Texas MD Anderson Cancer Center, Houston, Texas, United States of America; Karolinska Institutet, Sweden

## Abstract

**Background:**

The MitoChip v2.0 resequencing array is an array-based technique allowing for accurate and complete sequencing of the mitochondrial genome. No studies have investigated mitochondrial mutation in salivary gland adenoid cystic carcinomas.

**Methodology:**

The entire mitochondrial genome of 22 salivary gland adenoid cystic carcinomas (ACC) of salivary glands and matched leukocyte DNA was sequenced to determine the frequency and distribution of mitochondrial mutations in ACC tumors.

**Principal Findings:**

Seventeen of 22 ACCs (77%) carried mitochondrial mutations, ranging in number from 1 to 37 mutations. A disproportionate number of mutations occurred in the D-loop. Twelve of 17 tumors (70.6%) carried mutations resulting in amino acid changes of translated proteins. Nine of 17 tumors (52.9%) with a mutation carried an amino acid changing mutation in the nicotinamide adenine dinucleotide dehydrogenase (NADH) complex.

**Conclusions/Significance:**

Mitochondrial mutation is frequent in salivary ACCs. The high incidence of amino acid changing mutations implicates alterations in aerobic respiration in ACC carcinogenesis. D-loop mutations are of unclear significance, but may be associated with alterations in transcription or replication.

## Introduction

Adenoid cystic carcinoma (ACC) of the salivary gland is a rare malignancy, comprising 1% of all head and neck tumors, 10% of all salivary gland masses, and 22% of all salivary gland malignancies. Surgical therapy with adjuvant radiation is the mainstay of treatment. The clinical behavior of ACC is atypical with an almost 40% incidence of distant metastases despite adequate local control with surgery and external beam irradiation [1]. Other atypical clinical features of ACC include a variable survival in patients with metastatic disease in a site dependent fashion. Patients with isolated pulmonary metastases have a much longer survival relative to patients with bone involvement [2].

Many types of combination chemotherapy and molecular therapy have been investigated for use in ACC. Limited response has been demonstrated in any regimen which has been employed in a clinical trial to this point [3]. There are a number of newer trials in progress, but there have been no major breakthroughs in chemotherapy for ACC.

The basis of these disappointing results is a lack of understanding of the basic biologic mechanisms involved in ACC. Several preliminary studies have investigated DNA alterations of known proto-oncogenes and tumor suppressor genes in ACC [4]–[12]. However, a clear picture of the molecular underpinnings of this malignancy is not apparent. Cytogenetic studies of ACC have demonstrated a pattern of chromosomal alterations that are not consistent with known solid tumor types, suggesting that there may be different mechanisms involved in its carcinogenesis [13].

Recent studies of other solid tumors have investigated the incidence and role of somatic mitochondrial mutations and their implications for carcinogenesis [14]–[24]. The mitochondrial genome is a 16.6.kb double stranded circular DNA containing no introns, including 13 genes that encode proteins that are involved in aerobic respiration, 22 transfer RNAs, and 2 ribosomal RNAs. The mitochondrial genome contains limited DNA repair mechanisms and is susceptible to DNA damage acquired from both reactive oxygen species (ROS) generated by cellular metabolism and from extrinsic carcinogens including tobacco and other substances. This susceptibility to mutation can potentially cause generation of additional ROS due to impairment of oxidative phosphorylation, facilitating DNA damage to the nuclear or mitochondrial genome as well as dysregulation of apoptosis and other cellular processes [25].

The advent of sensitive oligonucleotide sequencing arrays has led to novel possibilities for molecular detection of cancer. Studies of a previous version of the MitoChip have proven its fidelity and reproducibility, and demonstrated the promise in application of mitochondrial sequencing in detection of tumor specific mitochondrial alterations [26]. Recently, a study by our group, utilizing a high throughput mitochondrial sequencing array for the entire mitochondrial genome has demonstrated a 49% of incidence of mitochondrial mutation in head and neck squamous cell carcinoma in another limited cohort [27]. A related study using the MitoChip identified mitochondrial mutations in premalignant gastrointestinal tract lesions [28].

No studies thus far have investigated mitochondrial DNA mutations in adenoid cystic carcinoma. A single report has demonstrated scattered mitochondria and a paucity of endoplasmic reticulum in adenoid cystic carcinoma derived from minor salivary glands [29], though this was only a histologic analysis. Due to the lack of understanding of the molecular mechanisms in carcinogenesis of ACC and the potential therapeutic implications of identification of novel therapeutic targets, this study was designed to investigate the incidence and distribution of mitochondrial mutation in ACC. We utilize a whole mitochondria sequencing array, the MitoChip v2.0 [27] to characterize the mutations that are present throughout the mitochondrial genome in 22 adenoid cystic carcinomas of the salivary glands in order to determine the prevalence and distribution of mitochondrial mutations in this disease.

## Materials and Methods

### Human Mitochondrial v2.0 Oligonucleotide Microarray

The MitoChip v2.0 was obtained from Affymetrix (commercially available GeneChip Human Mitochondrial Resequencing Array 2.0; Santa Clara, CA). Sequences comprising both strands of the entire human mitochondrial genome were synthesized as overlapping 25-mers on high-density oligonucleotide arrays with 8×8-µm features. To query any given site from the human mitochondrial reference sequence, four features are tiled on the MitoChip. The four features differ only by the 13th base, which consists of each of the four possible nucleotides.

### DNA Sample Source and Preparation

Tumor samples and leukocyte DNA were obtained from resected specimens of 22 patients with adenoid cystic carcinoma who undergone enrollment in institutional review board had approved protocols at Johns Hopkins Hospital and the University of Texas M.D. Anderson Cancer Center after informed consent was obtained. At the Johns Hopkins Hospital, tumors were collected under IRB #92-07-21-01, and at the M.D. Anderson Cancer Center, tumors were collected under IRB #Lab92-018. The research was performed at Johns Hopkins under the IRB #NA_00001336. Written informed consent was obtained from all those patients. Tumor specimens were snap frozen in liquid nitrogen and stored at −80 degrees Celsius. Samples were microdissected on a cryostat so that the tumor samples contained greater than 70% neoplastic cells. DNA from tumor sections was digested with 1% sodium dodecyl sulfate/proteinase K, extracted by phenol-chloroform, and ethanol precipitated. Control DNAs from peripheral lymphocytes were processed in the same manner as described previously [30]. Mitochondrial mutation status in these tumors were identified by sequencing of tumors and matched leukocyte DNA samples using the MitoChip v 2.0 mitochondrial resequencing array (Affymetrix; Santa Clara, CA) as previously described [27] by comparison of tumor sequence with matched leukocyte sequence, to identify true mutations rather than polymorphisms.

### Mitochip Preparation

The entire mitochondrial DNA sequence was amplified in three overlapping polymerase chain reactions using 50 ng of genomic DNA each [26]. The reagents, conditions, and purification procedures were accomplished as described previously [27]. These three sets of PCR primers were F1: 5′-ATA GGG GTC CCT TGA CCA CCA TCC TCC GT-3′ and R1: 5′-GAG CTG TGC CTA GGA CTC CAG CTC ATG CGC CG-3′, F2: 5′-CCG ACC GTT GAC TAT TCT CTA CAA ACC AC-3′ and R2: 5′-GAT CAG GAG AAC GTG GTT ACT AGC ACA GAG AG-3′, F3: 5′-CAT TCT CAT AAT CGC CCA CGG GCT TAC ATC C-3′ and R3: 5′-GTT CGC CTG TAA TAT TGA ACG TAG GTG CC-3′. Amplification was accomplished in 50-µl PCR reaction performed in thin-walled polypropylene plates using the high-fidelity TaKaRa LA Taq (TaKaRa Mirus Bio, Madison, WI). The PCR conditions are 94°C for 2 min, 30 cycles of 94°C for 15 sec, 68°C for 7 min, and then an extension step at 68°C for 12 min followed by holding at 4°C. As a control for PCR amplification and subsequent hybridization, a 7.5-kb plasmid DNA (Tag IQ-EX template) was amplified concomitantly with the test samples, using forward and reverse primers included in the CustomSeq control kit (Affymetrix, Inc.). The specificity of the reactions was confirmed by agarose gel electrophoresis. The PCR products were purified using QIAQuick PCR Clean up kit (Qiagen, Inc., Valencia, CA), and the resultant purified DNA was resuspended in 30 to 40 µl of EB buffer (Affymetrix, Inc.). The concentration of each purified PCR product was determined by NanoDrop (Thermo scientific). PCR products from three reactions were mixed in equal molar concentrations. The pooling, DNA fragmentation, labeling, and chip hybridization were performed according to Affymetrix CustomSeq Resequencing protocol instructions.

The pooled 35 ul of DNA fragments were then digested with DNase I (0.2 U of DNase I/µg DNA) for 15 minutes in a 50-µl reaction (Affymetrix, Cat. 900447). Samples were then incubated at 95°C for 15 minutes to inactivate DNase I. Fragmented DNA was labeled by adding 2.0 µl of GeneChip DNA labeling reagent and 3.4 µl of 30 U/µl terminal deoxynucleotidyl transferase (both from Affymetrix). The labeling conditions were 37°C 90 min and 95°C 15 min. Prehybridization, hybridization, washing, and scanning of the MitoChip were performed as described in the Affymetrix CustomSeq Resequencing protocol. The prehybridizations were performed for 15 minutes in 80-µl (for v2.0 chips) solution containing 3 mol/L tetramethylammonium chloride, 0.1% Tween 20, and 10 mmol/L Tris, pH 7.8. The GeneChips for human mitochondrial resequencing array 2.0 (Affymetrix, Cat. 900886) were hybridized for 16 hours at 48°C with 60 rpm rotation in a hybridization solution containing 3 mol/L tetramethylammonium chloride, 100 µg/ml herring sperm DNA, 500 µg/ml bovine serum albumin, 10 mmol/L Tris, pH 7.8, 0.01% Tween 20, and 200 pmol/L control oligo. The chips were then washed on the Affymetrix fluidics station using the preprogrammed CustomSeq Resequencing wash protocols.

### Automated Batch Analysis of Microarray Data

Analysis of microarray data for the v2.0 MitoChips was done using GeneChip® Sequence Analysis Software (GSEQ) v 4.0 (Affymetrix). GSEQ uses an objective statistical framework, based upon the ABACUS algorithm [31] to assign base calls to each position which meets quality criteria in the mitochondrial genome. The original ABACUS algorithm has been successfully applied for high-throughput variation detection in humans as well as for detection of mitochondrial sequence variations in human embryonic stem cells and head and neck cancers [27], [32].GSEQ software was used per manufacturer's instructions, with Genome model set to “diploid” and Quality Score Threshold set to “3” to maximize base call percentage and fidelity.

## Results

### Demographics

Tumor samples were derived from 22 patients who underwent resection of primary or locally recurrent lesions, and one sample was derived from nodal disease at the time of initial resection of ACC. Samples were collected at Johns Hopkins Hospital and M.D. Anderson Cancer Center between 1992 and 2005 in the operating room and immediately snap frozen with liquid nitrogen. Tissue sources utilized to acquire tumor DNA in this study and other demographic information is recorded in Table 1. Mean age of the patients was 55 years (Range 18–74). Mean follow-up time was 68.1 months (Range 7–190 months). Twenty-one of 22 patients (95.5%) received adjuvant radiation, and 14/22 patients (64%) developed recurrence after resection. One patient (Patient 5) presented with locally recurrent and metastatic disease after resection at another institution. Of the patients who recurred after resection at our institutions, median time to recurrence was 24 months (Range 1–93 months).

**Table 1:** Demographic and clinical patient data.

					Staging	_ _	Size			Followup	Lung		Total	D-loop	AA	NADH
Patient	Sex	Age	Ethnicity^1^	Location	T	N	M	(cm)	X-RT	DFI^2^	(Month)	Met^3^	LR^4^	Mut.	Mut.	Mut.	Complex
1	M	49	C	Maxilla	1	0	x	-	Y	-	161	−	−	1	1	0	0
2	F	61	AA	BOT^5^	2	0	x	-	Y	-	155	−	−	7	1	5	4
3	F	18	A	Maxilla	-	-	1	-	Y	93	167	+	+	0	0	0	0
4	F	43	C	BOT	4	2	x	6.5	Y	-	27	−	−	2	0	2	1
5	M	31	A	Parotid	-	-	1	-	Y	190	190	+	+	37	6	12	5
6	F	68	C	EAC^6^	1	0	0	0.5	Y	-	48	−	−	29	13	4	3
7	M	51	AA	Submandibular	2	0	0	-	N	18	65	+	+	11	1	1	0
8	F	73	C	Maxilla	3	0	x	4.6	Y	-	18	−	−	0	0	0	0
9	F	73	H	Parotid	2	0	x	3	Y	47	55	+	−	3	0	2	0
10	F	74	C	Parotid	2	0	x	3	Y	44	100	+	−	3	1	0	0
11	M	30	C	Parotid	4	2	1	15	Y	1	13	+	−	1	0	1	1
12	M	52	C	Parotid	2	0	x	3.7	Y	9	17	+	−	2	0	1	0
13	F	74	C	Parotid	4	0	x	7	Y	-	57	−	−	2	1	0	0
14	M	63	H	Parotid	2	0	x	4	Y	6	7	−	+	3	1	1	1
15	M	48	C	Parotid	3	0	x	5.5	Y	92	112	+	−	3	1	1	1
16	M	73	C	Parotid	3	0	x	6	Y	24	40	+	−	0	0	0	0
17	M	69	AA	BOT	2	0	x	2.5	Y	22	30	+	+	1	0	0	0
18	F	34	C	Maxilla	2	0	x	3.5	Y	11	27	+	−	0	0	0	0
19	F	67	H	BOT	3	0	x	4	Y	-	42	−	−	3	0	0	0
20	M	51	C	Submandibular	2	0	x	3.2	Y	-	42	−	−	6	1	4	1
21	F	73	C	Submandibular	2	0	x	2.5	Y	26	47	+	−	28	6	3	1
22	M	44	AA	BOT	3	0	x	6	Y	72	79	+	−	0	0	0	0

1 C: Caucasian, AA: African American, A: Asian, H: Hispanic.

2 DFI: disease free interval (Month).

3 Lung metastasis.

4 LR: local recurrence.

5 BOT: Base of Tongue.

6 EAC: External Auditory Canal.

### D-loop and NADH Complex Are Mutated in the Majority of Salivary Gland Adenoid Cystic Carcinomas

In total, 727,936 bases were sequenced from 44 samples comprised of DNA derived from 22 tumors and matched peripheral blood leukocytes to provide germ line mitochondrial sequence data. The median call rate of sequencing for each sample was 98.05% (Range 93.5%–99.9%). Mitochondrial mutations, defined as sequence differences between tumors and germ line DNA were identified in 17/22 (77%) samples tested. One hundred forty two mitochondrial mutations were identified (Table S1, range 1–37 mutations/tumor). D-loop mutations were identified in 11/22 (50%) tumors (Range 1–13 mutations/tumor), and 51 mutations were identified in NADH complex genes.

Thirty eight non-synonymous mutations were present among 12/22 (54.5%) tumors (Range 1–12 mutations) (Table 2). Twenty-two of 38 (57.8%) of non-synonymous mutations occurred in NADH complex genes, with 9/12 (75%) tumors carrying amino acid changing mutations demonstrating at least one mutation in NADH complex genes. Three of these tumors carried multiple mutations in NADH complex genes.

**Table 2:** Non-synonymous somatic mitochondrial DNA and amino acid alterations.

				mtDNA Mut.		Amino acid Mut.	
Patient	Position^1^	Gene	Reference^2^	Leukocyte	Tumor	Leukocyte	Tumor
2	9450	COIII	G	G	T	G	W
2	10371	ND3	G	G	A	E	K
2	10602	ND4L	A	A	G	T	A
2	10848	ND4	A	A	G	H	R
2	12650	ND5	T	T	C	L	P
4	9097	ATPase6	A	A	G	I	V
4	10967	ND4	A	A	G	T	A
5	3788	ND1	A	A	G	N	S
5	3789	ND1	C	C	G	N	K
5	5302	ND2	T	T	C	I	T
5	5910	COI	G	G	A	A	T
5	6391	COI	A	A	C	N	T
5	9053	ATPase6	G	G	A	S	N
5	9055	ATPase6	G	A	G	T	A
5	10398	ND3	A	G	A	A	T
5	10491	ND4L	A	A	G	N	Y
5	10609	ND4L	T	T	C	M	T
5	12406	ND5	G	G	A	V	I
5	13928	ND5	G	G	C	S	T
6	4216	ND1	T	C	T	H	Y
6	10398	ND3	A	G	A	A	T
6	12612	ND5	A	G	A	E	V
6	15257	Cytb	G	A	G	N	D
7	6112	COI	T	T	C	V	A
7	14577	ND6	T	T	C	I	N
9	9080	ATPase6	A	A	C	N	T
9	14786	Cytb	A	A	T	I	F
11	11202	ND4	A	A	C	Y	S
12	15837	Cytb	T	T	G	I	S
14	3614	ND1	T	T	G	L	R
15	5074	ND2	T	T	C	I	T
20	7641	COII	A	A	G	E	G
20	8249	COII	G	G	A	G	STOP
20	10576	ND4L	T	T	C	M	T
20	14758	Cytb	A	A	G	M	V
21	8075	COII	G	G	A	A	T
21	9055	ATP6	G	G	A	A	T
21	10398	ND3	A	A	G	T	A

1,2 based on GenBank Homo sapiens mitochondrial genome, NC_012920.

### Mutations Appear to be Distributed Nonrandomly Throughout the Mitochondrial Genome

Most mitochondrial mutations are spread throughout the genome with frequencies roughly proportional to the size of the gene in which they are found relative to the size of the mitochondrial genome (Table 3). However, the incidence of mutations in the D-loop is significantly higher than expected for a random distribution of mutations based upon the size of the region alone (23.2% of mutations vs 6.8% of total size of genome, p value<0.01, Chi Square Test).

**Table 3:** Proportion of mitochondrial DNA and amino acid changes to gene size.

Gene	Size (bp)	% of Genome	# of mtDNA Mut.	% of mtDNA Mut.	# of AA Mut.
12S Ribosomal RNA	953	5.8	3	2.1	-
16S Ribosomal RNA	1557	9.4	10	7.0	-
ATPase6	680	4.1	7	4.9	5
ATPase8	206	1.2	1	0.7	0
COI	1541	9.3	11	7.7	3
COII	701	4.2	7	4.9	3
COIII	783	4.7	3	2.1	1
Cytb	1140	6.9	8	5.6	4
D-Loop	1121	6.8	33	23.2	-
ND1	955	5.8	8	5.6	4
ND2	1041	6.3	5	3.5	2
ND3	345	2.1	6	4.2	4
ND4	1377	8.3	13	9.2	3
ND4L	296	1.8	7	4.9	4
ND5	1811	10.9	10	7.0	4
ND6	524	3.2	2	1.4	1
tRNA arginine	64	0.4	1	0.7	-
tRNA isoleucine	68	0.4	2	1.4	-
tRNA serine 1	70	0.4	1	0.7	-
tRNA alanine	68	0.4	1	0.7	-
tRNA asparagine	72	0.4	1	0.7	-
tRNA methionine	67	0.4	1	0.7	-
tRNA leucine 2	70	0.4	1	0.7	-
Total			142	100.0	

Utilizing bivariate analysis, no other statistically significant association was identified between the number, distribution, or nature (synonymous or non-synonymous) of mitochondrial mutations and metastases, recurrence, ethnicity, initial stage, or location of tumors.

## Discussion

In this study we have applied a high throughput approach to sequencing the entire mitochondrial genome to salivary gland ACC samples and compared these sequences to matched germ line samples in order to determine the incidence and nature of mitochondrial mutations in this disease. This report represents the first investigation of the frequency of mitochondrial mutation in this disease. Additionally, we have identified a region of the mitochondrial genome, the D-loop which is selectively mutated in ACC at a rate that is much higher than the remainder of the genome. The majority of tumors with amino acid changing mutations demonstrated a mutation in the ND complex.

The high incidence of amino acid changing mutations in the ND complex and observation of a propensity for accumulation of multiple mutations once mitochondrial damage is initiated is consistent with a role of mitochondrial mutation as a potentiator of DNA damage in ACC. Previous studies from our group have shown ND complex mutations, particularly, the ND2 mutation, is accompanied by increased ROS generation and hypoxia inducible factor-a (HIF-1α) stabilization and anchorage independent growth [33]. Another study has implicated ND6 mutation with dysregulation of response to hypoxia in glioma cells [34]. Sequential mutation of the ND complex in ACC may result in a spiraling cycle of increased ROS production and dysfunction of the aerobic glycolytic mechanism with subsequently increased propensity for further mutation and dysfunction resulting in a competitive growth advantage for tumor cells.

Mitochondrial mutations in several gene complexes have been shown to contribute to tumorigenicity through ROS generation in several tumor types [33], [35], [36]. A recent study has demonstrated conference of metastatic potential by ND1 complex mutation through ROS generation in a tumor specific fashion [37]. This parallels the finding of worsening prognosis with increased mitochondrial mutation cervical cancer [38]. While we did not detect an association between mitochondrial mutation and the clinical behavior of ACC, larger studies may need to be performed to adequately address this question.

We made a comparison of our reported somatic mitochondrial nonsynonymous mutations to the mitochondrial genome database [39]. Several non-synonymous mutations that were reported previously might have functional correlations with human disease. In our study, we found two out of 22 cases with G10398A mutation in ND3, while one patient had the reverse alteration detected (A10398G). G10398A had been associated with invasive breast cancer and esophageal cancer in African American individuals[40]–[42]. However, there is also conflicting evidence to suggest that this is not the case[43]. Interesting, the reverse finding (A10398G mutation) was detected in thyroid carcinoma as well [23]. Another mutation, T4216C found in ND1 in patient #6, was suggested to increase risk of insulin-resistance and type 2 diabetes [44]. Evidence also supports its association with Leber hereditary optic neuropathy [45]. T14577C mutation in ND6 was also implicated as the pathogenic mutation for maternally inherited type 2 diabetes [46].

The fact that there were similar somatic mutations detected in our ACC cohort as reported in other tumor types raises the possibility that these mutations in particular might play a role in positively selecting for these cancer cells beyond random mutation. Zhidkov et al [47] suggest that these alterations form a pattern of mutation that can be traced to ancient phylogeny. This finding further implicates these mutations as being functionally relevant as they have persisted through evolutionary pressures.

The mitochondrial D-loop is an approximately 1kB region of the mitochondrial genome which contains *cis*-acting elements involved in regulation of transcription and replication of the mitochondrial genome [48]. The exact contribution of the D-loop to carcinogenesis is poorly understood, however, mutation of the D-loop has been associated with alterations in mitochondrial copy number in a variety of solid tumors [49]–[51]. Our findings of disproportionate degree of mutation of the D-loop are consistent with previous studies showing alterations in regulation of mitochondrial transcription and replication may contribute to carcinogenesis in ACC.

Identification of mitochondrial mutations in ACC has important implications for disease detection and therapy. The relative chemoresistance of this tumor type makes identification of novel therapeutic targets imperative to improvement of treatment for this disease. Amino acid changing mutations of the complexes regulating oxidative phosphorylation carry immunologic implications as well as functional implications [52]. Further studies are warranted, but it seems feasible that alterations in immunogenicity are inherent with the mutations which have been identified. This may provide additional, disease specific, targets for immunotherapy in ACC.

Application of the quantitative nature of the techniques employed in this study may be useful for detection of tumor specific mitochondrial mutations as an adjunctive study in margin analysis. Previous studies by our group have shown that the MitoChip can be successfully employed to detect tumor specific mitochondrial mutations in a background of normal DNA [33]. The high incidence of local and regional recurrence despite complete resection as assessed by conventional pathologic assessment demonstrates the need for development of molecular techniques to evaluate margins in this disease.

One of the limitations of this study is that we have a relatively small cohort of ACC due to the rare nature of this malignancy, with approximately 600 new cases diagnosed each year in the US. Future studies with larger sample size would be beneficial to better understand the relationship of somatic mitochondrial mutations and ACC.

In this study we have employed a genome wide sequencing technique to identify mitochondrial mutations in adenoid cystic carcinoma. We have reported a high incidence of mitochondrial mutation in ACC, a novel finding. Further investigation is warranted as to the functional implications of these mutations in carcinogenesis. However, these findings and the technique utilized to identify them has significant potential for novel detection methods and therapy in this disease.

## Supporting Information

Table S1 Summary of somatic mitochondrial mutations in ACC
(0.04 MB XLS)
